# High adherence and low dropout rate in a virtual clinical study of atopic dermatitis through weekly reward-based personalized genetic lifestyle reports

**DOI:** 10.1371/journal.pone.0235500

**Published:** 2020-07-02

**Authors:** Zarqa Ali, Kathryn Anderson, Andrei Chiriac, Anders Daniel Andersen, Ari Pall Isberg, Fernando Gesto Moreno, Aleksander Eiken, Simon Francis Thomsen, John Robert Zibert

**Affiliations:** 1 Department of Dermatology and Wound Healing Centre, Bispebjerg Hospital, Copenhagen, Denmark; 2 LEO Innovation Lab, Copenhagen, Denmark; 3 Department of Biomedical Sciences, University of Copenhagen, Copenhagen, Denmark; University and University Hospital Basel, SWITZERLAND

## Abstract

**Introduction:**

Clinical trials often suffer from significant recruitment barriers, poor adherence, and dropouts, which increase costs and negatively affect trial outcomes. The aim of this study was to examine whether making it virtual and reward-based would enable nationwide recruitment, identify patients with variable disease severity, achieve high adherence, and reduce dropouts.

**Methods:**

In a siteless, virtual feasibility study, individuals with atopic dermatitis (AD) were recruited online. During the 8-week study, subjects used their smartphones weekly to photograph target AD lesions, and completed patient-oriented eczema measure (POEM) and treatment use questionnaires. In return, subjects were rewarded every week with personalized lifestyle reports based on their DNA.

**Results:**

Over the course of the 11 day recruitment period, 164 (82% women and 18% men) filled in the form to participate, of which 65 fulfilled the inclusion criteria and signed the informed consent. Ten were excluded as they did not complete the mandatory study task of returning the DNA sample. 55 (91% women, 9% men) subjects returned the DNA sample and were enrolled throughout Denmark, the majority outside the Copenhagen capital region in rural areas with relatively low physician coverage. The mean age was 28.5 (SD ±9.5 years, range 18–52 years). The baseline POEM score was 14.5±5.6 (range 6–28). Based on the POEM, 7 individuals had mild, 28 had moderate, 17 had severe, and 3 had very severe eczema. The retention rate was 96% as 53 out of 55 enrolled completed the study. The adherence was very high, and more than 90% of all study tasks were completed. Follow up of 41 subjects showed that 90% would take part again or continue if the study had been longer.

**Conclusion:**

A virtual trial design enables recruitment with broad geographic reach and throughout the full spectrum of disease severity. Providing personalized genetic reports as a reward seems to contribute to high adherence and retention.

## Introduction

Recruitment, adherence, and retention are critical to the success of clinical trials. Failing to enroll a sufficient number of subjects in a trial is a continuing problem [1]. A review of 114 trials in the UK showed that only 31% met enrollment goals [2]. Campbell et al. [3] reported that one-third of publicly funded trials failed to meet initial recruitment goals and required a time extension. The willingness of the participant to travel to and from the local study center affects recruitment [4], and long travel times can dissuade participation and result in recruitment from only proximate vicinities of the study centers. Further, digital techniques such as web-based screening have increased recruitment efficiency rates [5,6]. However, the effect of this on adherence and retention rates is not well understood. Adherence in a clinical trial is defined as the extent to which the participant’s behavior coincides with the trial protocol in terms of keeping clinic appointments, taking medication, and completing trial tasks. Adherence in clinical trials is a consistent problem and often results in high dropout rates [7]. Dropouts threaten the internal and external validity of results, as completers may differ from dropouts. A review of 71 randomised controlled trials in four highly cited medical journals showed dropout rates of 20% or more [8]. The consequence of missing data is a reduction in statistical power to detect treatment effects, possibly biasing trial conclusions.

Atopic dermatitis (AD) is an inflammatory, chronically relapsing, and intensely pruritic skin disease characterized by dry skin [9]. The lifetime prevalence of AD is around 20% [10] and it is the highest ranked skin disease with respect to disability-adjusted life-years and years lived with a disease [11].

Given the significant problem of clinical trials that fail to succeed due to poor recruitment, enrollment, adherence, and retention, it is of paramount importance to identify procedures that eliminate or minimize these issues. The aim of this study was to investigate the feasibility of a virtual trial, i.e. whether a patient-centric, siteless, reward-based, remote trial of patients with AD can 1) recruit nationwide, 2) identify patients with variable disease severity, 3) achieve high adherence, and 4) prevent dropouts.

## Methods

### Screening

This was a non-interventional, siteless, virtual feasibility study, recruiting AD subjects online. Study participants were recruited through advertisements on Facebook. The advertisements were shown to subjects searching for or “liking” skin products or AD-relevant Facebook content. By clicking on the advertisements, potential participants were redirected to a website where they were able to sign up for the study. Study eligibility was evaluated as per the inclusion/exclusion criteria blinded by an in-house dermatologist. The inclusion criteria were signed informed consent, age of 18 years or older, AD meeting the UK Diagnostic Criteria for Atopic Dermatitis [12], at least one visible AD lesion at the time of recruitment (as confirmed remotely from photographs uploaded during screening), smartphone with a functioning camera, willing to donate a saliva sample for DNA analysis, and confirmed intention to comply with study protocol procedures. The exclusion criteria were female subjects who were pregnant (or planned to become so during the study period) or lactating, active dermatological condition that may interfere with the diagnosis of AD and/or the assessment of disease activity, inability to speak or understand Danish, and no visible AD at time of screening.

^Informed consent was obtained online. After consent was obtained, subjects were asked to download the app “Imagine Skin Tracker” (^^https://getimagine.io/^^, LEO Innovation Lab, Copenhagen, Denmark). “Imagine Skin Tracker” is a smartphone application compatible with all smartphone platforms, and was developed to help patients track skin lesions over time. The app further enables dermatologists to evaluate these photographs. In this study the participants used the “Imagine Skin Tracker” App to take photographs of their skin lesions. Questionnaires were sent separately to participants as Google Forms. Each week participants received an email prompting them to complete tasks, containing a link to the questionnaire and a reminder to use “Imagine Skin Tracker”, and these emails served as the primary means of communication between study staff and the participants.^

### Enrollment

Enrolled subjects, who had signed the informed consent, received a DNA collection kit by post and were asked to provide a saliva sample for DNA extraction. To ensure correct sampling, subjects could contact the study team by phone if needed. Upon sampling, the subjects shipped the tube containing their saliva sample (which was de-identified with a barcode) directly to the laboratory using a prepaid envelope. Providing a saliva sample for DNA extraction was a mandatory study task to continue the study as it was used to generate DNA lifestyle reports which were used as a retention strategy to reward the participants after completing study tasks each week.

### Study design

Two study tasks were planned for each week throughout the 8-week study period. The subjects filled in online Patient-Oriented Eczema Measure (POEM) [13] and treatment use questionnaires each week. Furthermore, once a week using the “Imagine Skin Tracker” app they captured and uploaded photographs of skin lesions from three different anatomical regions selected by the subjects themselves in Week 0. When questionnaires and photographs were successfully submitted, the subjects received a personalized DNA lifestyle report as a reward for completing the weekly study task. Participants were prompted to complete study tasks each week by an email. This email contained two prompts, one asking them to open the Imagine app to take photographs, and the second was a link to a Google Form with the weekly questionnaire. Both could be carried out via the participants’ own smartphone.

^Compliance with study protocol (taking photos and filling out questionnaires) was checked manually by dedicated study staff. For all subjects, push notifications through automated email (Intercom, CA, USA (^^https://www.intercom.com/^^)) were used to prompt subjects to comply with the weekly study activities. If the participants failed to do so, reminders through text messages to participants’ mobile phones were sent manually as necessary. Repeated failure (defined as three weeks in a row) to comply with protocol procedures was considered a dropout. Completers were asked to fill in a feedback questionnaire after the 8 weeks study period.^

### Personalized DNA lifestyle reports

DNA sample collection was performed once in the beginning of the study as a mandatory study task to continue the study. The genetic testing was a standard SNP microarray (Illumina global screening array, Illumina Inc.. CA, USA). This is a commercial service that is routinely used for GWAS and research. The DNA sample was used to generate 8 DNA lifestyle reports that were unlocked each week as a reward for completing study tasks. The SNPs and the content utilized for the reports were based on a rigorous systematic review of the scientific literature. In addition the language used in the reports reflected the fact that DNA results were based on association studies and statistical probability. The DNA lifestyle reports covered the following topics: Alcohol, Caffeine, Gluten, Injuries, Vitamin D, and Healthy weight.

### Ethics

Danish Regional Committee on Health Research Ethics was consulted prior to execution and the study did not require ethical approval (protocol number:16025688). The study was carried out according to Danish law. Participants were not offered any monetary incentives for participating in the study.

### Statistics

Data analyses were performed using R [14]. Baseline variables were reported as means and standard deviations for continuous variables and frequencies and percentages for categorical variables. Exploring the differences between dropouts and completers, the continuous variables were analyzed using the two-tailed Student t-test and binary variables were analyzed by the Chi square test. A p-value < 0.05 was considered statistically significant. A Kaplan-Meier survival graph was applied to visualize dropouts over time using the *survival* package in R [15]. The conversion rate was calculated by dividing the number of individuals signing up for the study by the number of individuals who visited the recruitment website and clicked on the button to participate. Recruitment rate was calculated by dividing the number of individuals included in the study by the recruitment period in days.

## Results

**Fig 1:**
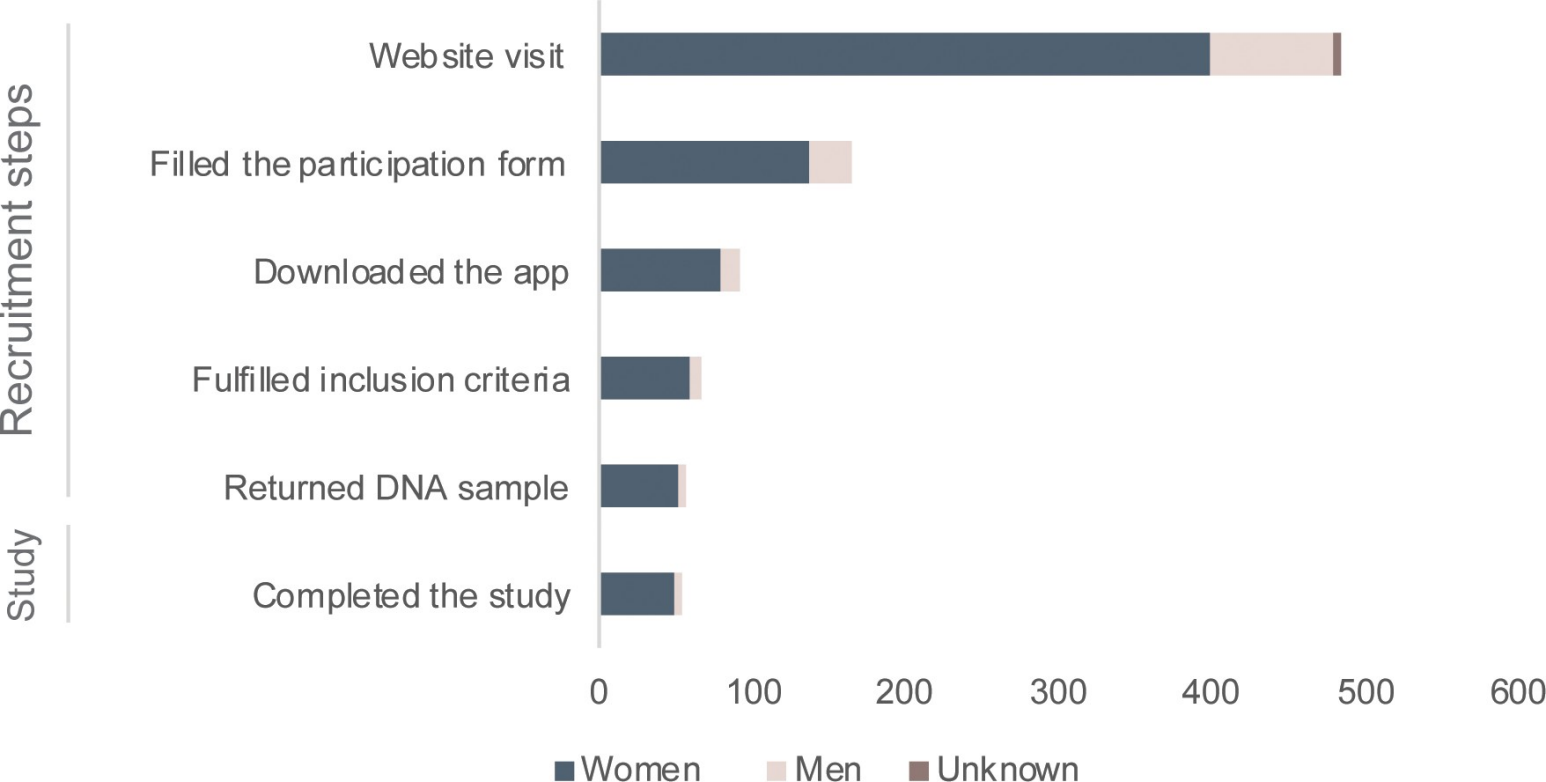
An overview of the numbers of participants and their gender at different steps of the recruitment.

The advertisements were live for 7 days during a period of 11 days, from March 17th and March 27th 2019. During these 7 days, the advertisements were seen by 172,683 individuals (75% women, 24% men, and 1% unknown), of which 481 (82% women, 17% men, and 1% unknown) visited the recruitment website and clicked on the button to participate. A total of 164 (82% women and 18% men) filled-in the form to participate, and of these, 91 (55%) downloaded the Imagine app and took a photo of a skin lesion as required (Fig 1). The photos were evaluated by an in-house blinded dermatologist and 65 applicants (71%) fulfilled the inclusion criteria and were enrolled. For the eligible subjects, it took on average 7 days to onboard participants into the study, from when they completed the form to when the DNA kit was sent to the subjects’ homes. A total of 55 returned the kit successfully and could continue the study. The average time taken from when the kit was sent, to when the kit was received by the laboratory was 9 days (including the saliva sample collection by the subject). The conversion rate was 14% (65/481). The recruitment rate per day was 9.3 (65/7), and 5.9 (65/11) for the period the ad was online and for the entire recruitment period, respectively.

**Table 1:** Demographics and baseline characteristics of the participants (n = 55).

Characteristics	Participants (n = 55)
Gender, female / male	50 (91%) / 5 (9%)
Age, year	28.5 ± 9.5 (18–52)
POEM, baseline	14.5 ± 5.6 (6–28)
Mild	7 (13%)
Moderate	28 (51%)
Severe	17 (31%)
Very severe	3 (5%)
Asthma or allergy	41 (75%)

Data are given as mean ± SD with range in brackets, or as numbers with percentages in brackets.

**Fig 2:**
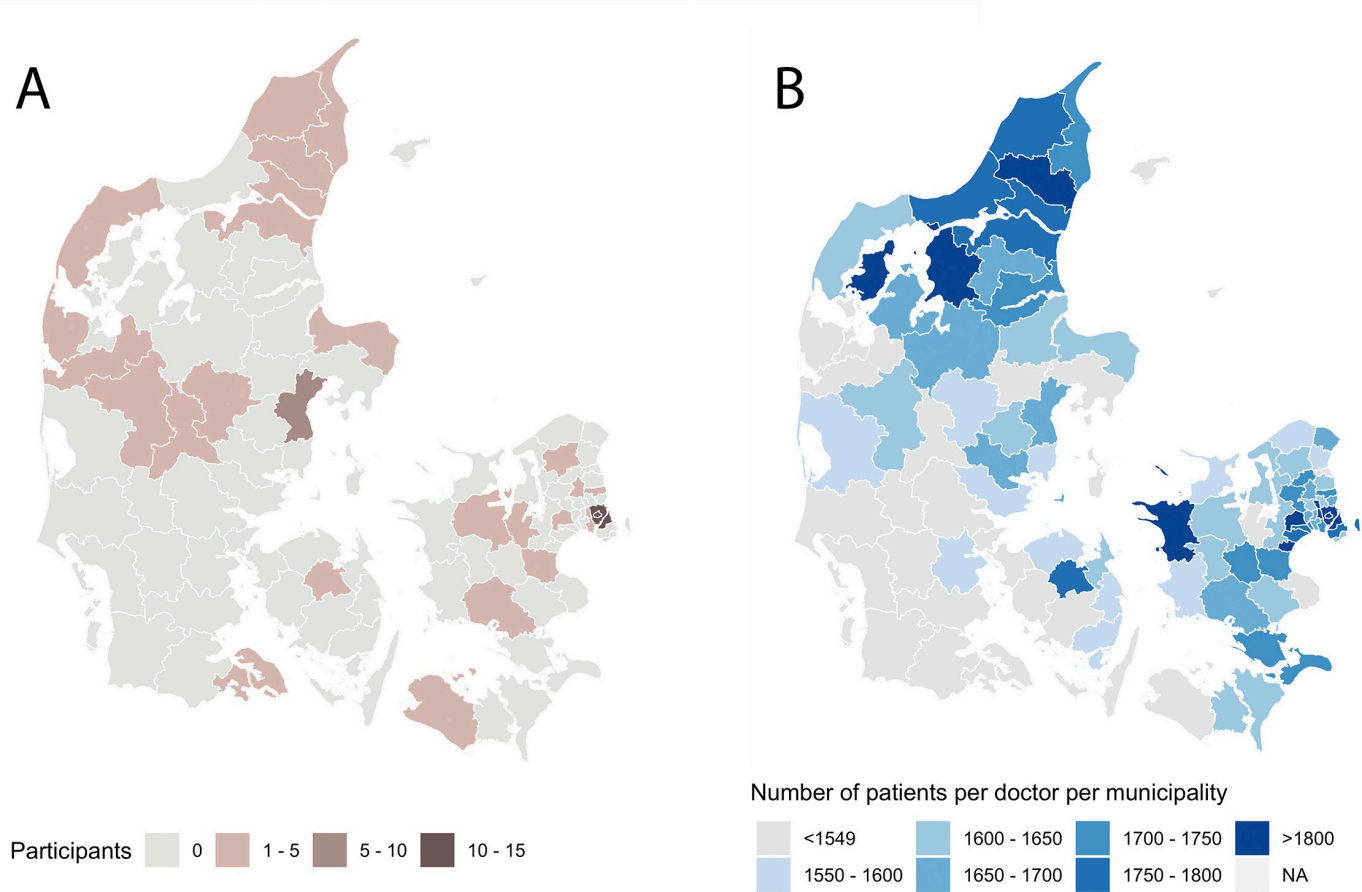
A) Recruitment locations in Denmark for the 55 participants enrolled in the study. B) A map showing municipalities with high numbers of doctors per inhabitant in Denmark (right), adapted from publicly available data [30]. Original map image source iStock.com/Rainer Lesniewski.

**Fig 3:**
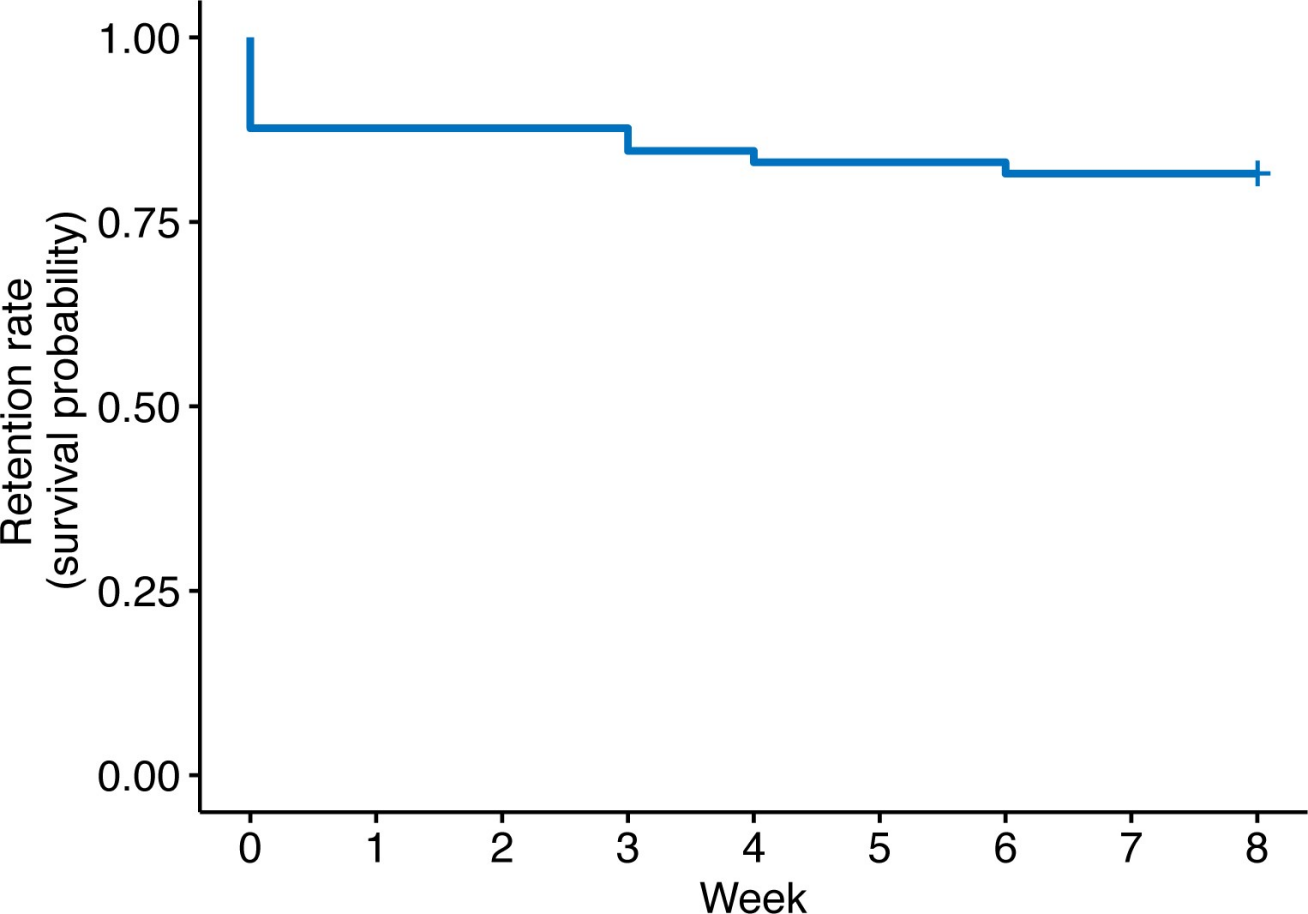
Kaplan–Meier survival curve showing the drop-out rate for the 65 participants who signed the informed consent.

The participants were enrolled throughout Denmark. The majority were enrolled from outside the Copenhagen capital region in rural areas with relatively low physician coverage (Fig 2). Ten participants signed the informed consent but did not complete the mandatory task of returning the DNA sample. This gave the largest drop in the number of participants in the beginning of the study (Fig 3). Participants who did not return their DNA sample were not retained in the study and were excluded. Of the 55 continuing in the study fifty were women (91%) and five were men (9%) (Table 1). Concomitant asthma or allergy was reported as 75% (when asked “Do you suffer from any types of allergies or asthma?” 41 said yes, 12 no, 2 don’t know/other). Participants reported using a range of treatments, such as moisturizers (87%), topical corticosteroids (80%), systemic treatments such methotrexate, corticosteroids etc. (11%), and UVA, UVB, or PUVA light therapy (15%). Around 15% of participants used other treatment methods, and most used more than one treatment method (76%), with 7% using four or more different methods.

**Fig 4:**
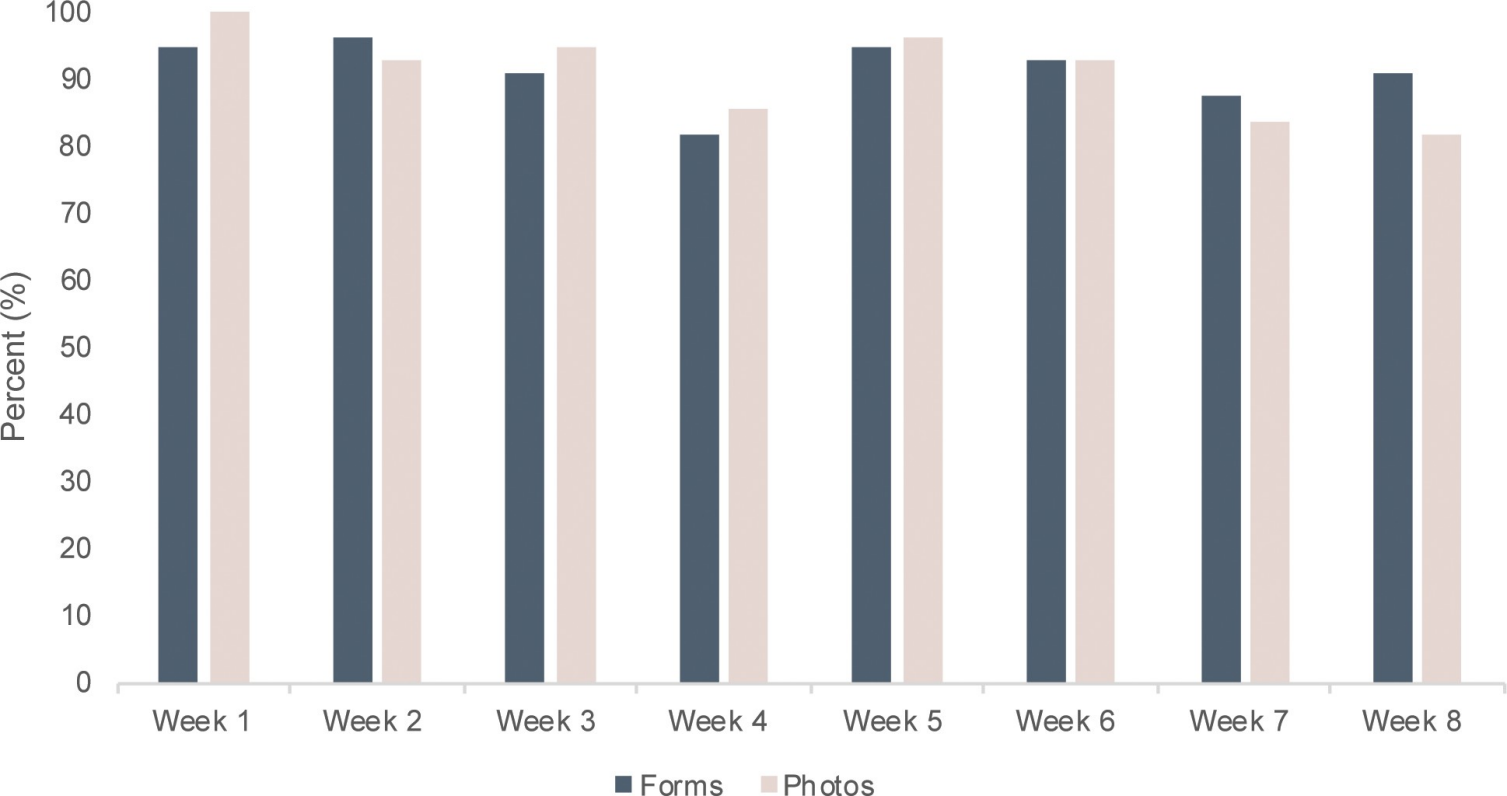
The percentage of participants completing study tasks of capturing photographs and completing questionnaires each week throughout the 8-week study period, N = 55.

The retention rate was 96%, as 53 (48 women and 5 men) out of 55 subjects completed the study. In the group of 65 (with the excluded participants) the retention rate was 82%. There were no significant differences between dropouts and completers regarding age, gender, and POEM. The adherence, for those continuing in the study (n = 55), was more than 90%, and out of a possible 880 (eight photographs and eight questionnaires for 55 participants) study tasks, 794 were completed (Fig 4). More than 52% of participants (N = 29) completed all of their assigned study tasks i.e. capturing photographs of AD lesions and completing the questionnaire every week. Of the 8 weeks of questionnaire tasks (not including photograph tasks), 66% of participants completed all 8, 11% completed 7, and 23% completed 6 tasks or less. While these numbers were similar for the weekly photo tasks, the proportion of participants completing all 8 tasks was lower (53%).

### Satisfaction with the study

Out of the 53 participants that completed the study, 41 answered the feedback questionnaires after the study was completed.

**Fig 5:**
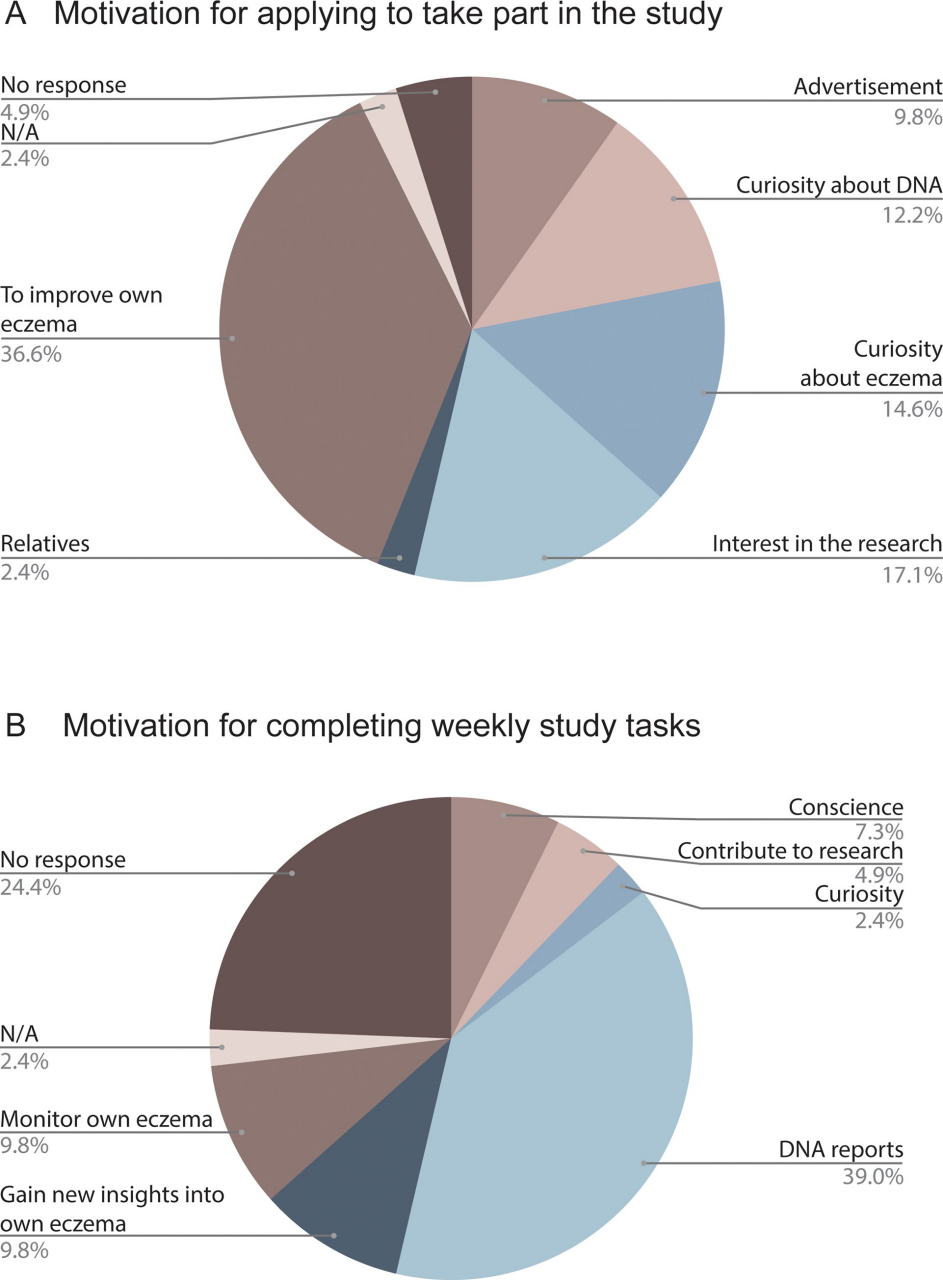
Distribution of self-reported motivation for taking part in the study. A) Motivation for applying to take part in the study answered by 41 enrolled participants, adapted into 5 categorizations based on free text answers. B) Motivation for completing the weekly tasks (photographs and questionnaires).

The most common reason given for applying to participate in the study was to improve their own eczema (37%, N = 15) (Fig 5A), with the second most common reason being interest in the research (17%, N = 7). The most common motivating factor given for completing weekly study tasks was the DNA reports (39%, N = 16), while 20% (N = 8) were motivated by interest in their own skin condition, and 7% (N = 3) were motivated by curiosity or contribution to research (Fig 5B).

**Fig 6:**
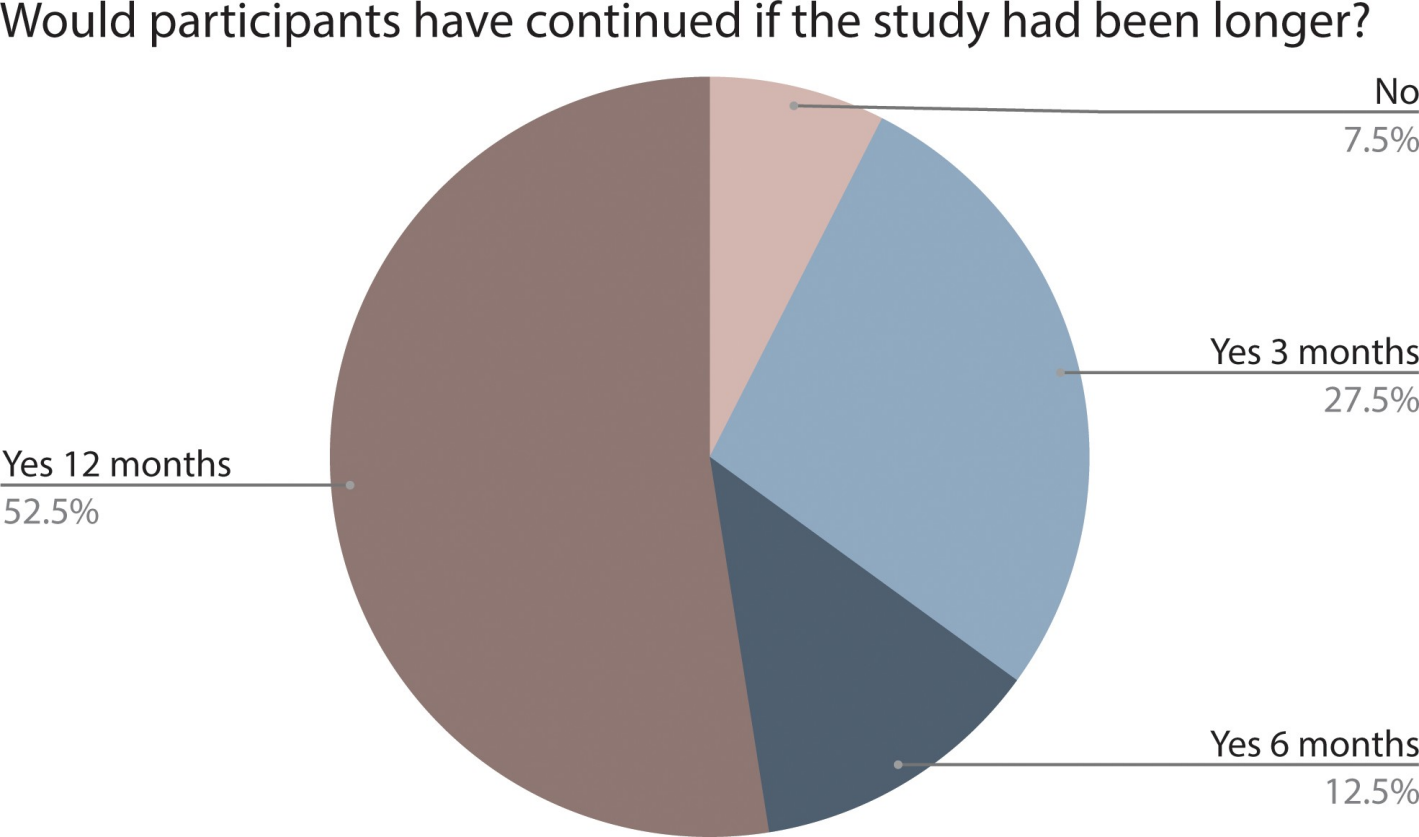
The distribution of how many participants would have continued the study and for how long (N = 41).

The majority (59%, N = 24) of the participants found the DNA reports exciting, and the reports were read completely or almost completely by 78% (N = 32). More than 95% (N = 39) felt that the DNA reports matched their own life experience to some degree or most of it. Ninety percent of participants indicated that they would have continued if the study had been longer (Fig 6), 90% (N = 37) were willing to participate in a similar study again, though it should be mentioned that self-reporting of willingness to continue a study does not necessarily predict future participation. All responding participants (N = 41) felt comfortable with sharing their DNA data with the study team for research purposes.

## Discussion

Retention rate is a key element in assessing the quality of a clinical trial. The retention rate in the present study was 96%; much higher than the average retention rate of about 80% in clinical studies with high retention rates [16]. A high attrition rate increases the risk of bias and lower compliance with study tasks [17]. In clinical trials poor compliance can threaten the outcome by reducing the power. Inadequate compliance by 30% of the participants can, under certain conditions, double the number of participants needed to produce a study with the same α and β values [18,19]. The adherence in the present study was measured by weekly questionnaires and photographs of targeted lesions. Several studies have shown that digital/online diaries improve the compliance significantly compared to traditional paper diaries; however, they still fall short of desired compliance levels [20]. Another study showed that use of rewards in mobile games increases compliance compared to electronic participant-reported outcome diaries without in-game rewards [21]. The adherence was very high in the present study and more than 90% completed the weekly tasks. Between the two study tasks, more participants managed to complete all 8 questionnaires than all 8 photo tasks (66% versus 53%), indicating an area of possible friction which could be improved in future studies, for example through integrating multiple apps into one platform.

The DNA reports were used as a reward for complying with the requirements of the participant tasks in the study protocol. More than 50% of the participants reported they were motivated to complete the weekly tasks due to the subsequent unlocking of the DNA reports. Furthermore, reminders to complete study tasks were also used as part of the retention strategy. Finally, we believe that the ease of participating in the study also had a remarkable impact on the retention rate as the participants could complete their study tasks from home at a suitable time. There were no obstacles such as transportation or waiting times at the study site. The dedication to the study was also reflected in the fast enrollment. It only took 9 days, on average, from the DNA kit being sent from the office to it being received at the laboratory. Even though the DNA kit was shipped with express delivery it reflects the commitment from the participants as they were quick to take the sample and return it.

Studies have documented that predictors for recruitment to and participation in clinical trials are ethnicity, income level, education level, age, and geographical area [22]. Studies conducted virtually could arguably influence some of those predictors. The majority of the enrolled participants in the present study were from outside the Copenhagen capital region from rural areas with relatively low physician coverage. Virtual trials may engage patients that normally would not have participated in traditional trials, and make nationwide recruitment possible. The elderly, disabled, those with mobility issues or those having difficulty traveling can participate in virtual trials without the discomfort associated with traveling. Young people of working age can participate in virtual trials, in contrast to traditional trials where those who had the time commitment were significantly more likely to participate [23]. Further, college graduates, individuals with higher levels of education, and those with incomes of $100,000 or more, are more likely to receive information about clinical trials from the print media [23]. As a consequence of the broader recruitment due to online strategies, a more diverse patient population may also be more representative than what is normally seen in traditional clinical trials.

A review of 151 randomized clinical trials from the UK's National Institute for Health Research showed that the median recruitment rate was found to be 0.92 patients recruited per center per month and the largest recruitment rates ranged from 16 to 58 patients per center per month [24]. In the present study 55 were recruited in less than 1 month. Online recruitment is the fastest growing method of recruitment today and has advantages over traditional recruitment in terms of cost, reach, and time-saving. In the present study, Facebook was used to recruit participants.

Facebook has become a major player in the field of digital advertising and allows advertisers to create target audiences by specifying gender, marital status, age, and geographic region as well as other personal characteristics. In the present study the advertisement on Facebook was targeted to subjects “liking” AD pages, pages about skin products and subject searching for topics related to AD and skin products. Around 75% of those who saw the advertisement were women, this number was also reflected in the enrolled group. Seemingly, women are more proactive than men in addressing medical issues and engaging with health related content on Facebook. Further, there is also a distortion in the genders searching for skin products, and therefore an increased cost associated with reaching and recruiting men, in addition to a selection bias in how the advertisement was designed to be more visible for women than men. Going forward, more provision should be made for a more gender-balanced, and therefore representative, recruitment. The experience from the present study can be used specifically to tailor advertisements towards specific patient groups and genders according to the demands of the trial.

The current study has some limitations. First, the conversion rate in the present study is 14%, it may sound low, but it is actually high in online recruitment and above 5% is considered high (25% percentile) (Ref. https://www.wordstream.com/blog/ws/2014/03/17/what-is-a-good-conversion-rate). In an offline setting non-converters are the individuals who read the advertisement in a newspaper without signing up for the study, or those who stop by an advertisement poster and read it without signing up. These numbers cannot be easily calculated for offline recruitment. Studies have shown that online recruitment methods are superior to offline methods in terms of efficiency (total number of participants enrolled) and cost (the average cost per recruited participant is lower for online recruitment) [25]. Online recruiting reaches a much larger and more targeted audience than other methods [26]. Secondly, as a prerequisite of participating in this study the patient must own a smartphone and a Facebook account which may exclude some, especially the elderly, from participating. The majority of the enrolled participants in this study were young women. Among other things, the skewed distribution is probably due to the fact that the advertisement targeted subjects searching for or “liking” skin products. Even though studies have shown that the age of adults using Facebook is 18–65 years (mean 48.2 years) [27], future studies using online recruitment should be aware of this and consider advertising through other channels than via social media. For example, using websites of patient associations or pharmacies to target the elderly, to reduce bias in recruitment. Having a smartphone that can upload photographs is crucial for remote trials in dermatology. The IMD World Digital Competitiveness Ranking 2019, researched that Danes are likely to become the most advanced country in “e-participation”, and ranks currently number 7 in “smartphone possession” which holds great promise to be able to reach patient segments we didn’t reach in this study in future virtual study activities [28]. Further, Four out of five Danes ages 16–74 have an active profile on social media [29]. What is also important to highlight is the fact that we are likely to reach many of those patients that due to the cosmetic nature of the disease live in isolation, and hence reach those that are not visiting the classical route of recruitment through hospitals and private practice.

Thirdly, it should be mentioned that the present study is a short-term observational study with a limited number of weekly study tasks and this may contribute to the high retention rate. Future studies should investigate retention rate in randomised-controlled trials in a virtual setting with a control group.

In conclusion, a virtual trial design enables online recruitment with broad geographic reach in a short time and with high variability in disease severity. Further, providing personalized genetic reports as a reward seems to contribute to high adherence and retention. Larger decentralized studies are needed to investigate the feasibility of virtual trials.

## Supporting information

S1 Data (PDF)

S2 Data (PDF)

S3 Data (PDF)

S1 Raw data (XLSX)
