# Correction: High adherence and low dropout rate in a virtual clinical study of atopic dermatitis through weekly reward-based personalized genetic lifestyle reports

**DOI:** 10.1371/journal.pone.0237824

**Published:** 2020-08-12

**Authors:** The PLOS ONE Staff

## Notice of republication

An incorrect version of S1 Data was published in error. The publisher apologizes for the error. This article was republished on July 20, 2020 to correct for this error. Please download this article again to view the correct version.
