# Supplementary material for: High adherence and low dropout rate in a virtual clinical study of atopic dermatitis through weekly reward-based personalized genetic lifestyle reports
Source: PLoS One. 2020 Jul 2;15(7):e0235500. doi: 10.1371/journal.pone.0235500 (PMC7332076; doi:10.1371/journal.pone.0235500)

High adherence and low dropout rate in a virtual clinical study of atopic dermatitis through weekly reward-based personalized genetic lifestyle reports

Ari Pall Isberg

04-April-2020

Number of patients: 65. Number of patients not completing: 12

Retention rate, Kepler Meier graph

```
## Call: survfit(formula = Surv(time_dropped_out, missing_both) ~ 1, data = surv_data)
##
##  time n.risk n.event survival std.err lower 95% CI upper 95% CI
##     0     65       8    0.877  0.0407        0.801        0.961
##     3     57       2    0.846  0.0448        0.763        0.939
##     4     55       1    0.831  0.0465        0.744        0.927
##     6     54       1    0.815  0.0481        0.726        0.915
```

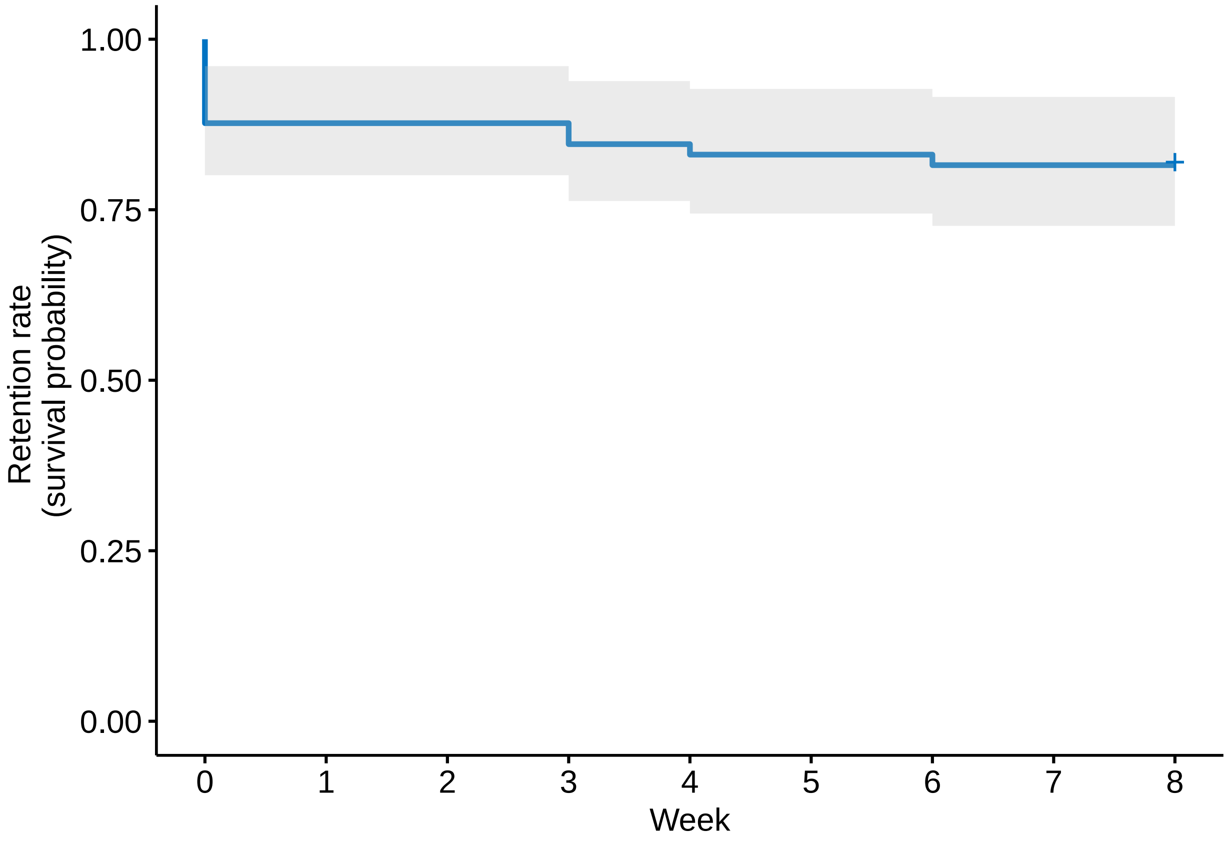

Visualize adherence

Bar plot 65 patients

Name of variables

- sum_form: Number tasks completed for forms for a given week
- sum_pic: Number tasks completed for pics for a given week
- n: Number of tasks available for form OR pic
- sum_all: Number tasks completed for pics and forms for a given week
- n_all: Number of tasks available for form AND pic
- perc_form: sum_form/n
- perc_pic: sum_pic/n
- cumsum_form: Cumulative sum for sum_form
- cumsum_pic: Cumulative sum for sum_pic
- cumsum_n: Cumulative sum for n
- cumsum_all: Cumulative sum for sum_all
- cumsum_n_all: Cumulative sum for n_all
- cumperc_form: cumsum_form/cumsum_n
- cumperc_pic: cumsum_pic/cumsum_n
- cumperc_all: cumsum_all/cumsum_n_all

variable	week_1	week_2	week_3	week_4	week_5	week_6	week_7	week_8
sum_form	56	54	50	45	52	51	48	50
sum_pic	58	52	52	47	53	51	46	45
n	65	65	65	65	65	65	65	65
sum_all	114	106	102	92	105	102	94	95
n_all	130	130	130	130	130	130	130	130
perc_form	0.862	0.831	0.769	0.692	0.8	0.785	0.738	0.769
perc_pic	0.892	0.8	0.8	0.723	0.815	0.785	0.708	0.692
cumsum_form	56	110	160	205	257	308	356	406
cumsum_pic	58	110	162	209	262	313	359	404
cumsum_n	65	130	195	260	325	390	455	520
cumsum_all	114	220	322	414	519	621	715	810
cumsum_n_all	130	260	390	520	650	780	910	1040
cumperc_form	0.862	0.846	0.821	0.788	0.791	0.79	0.782	0.781
cumperc_pic	0.892	0.846	0.831	0.804	0.806	0.803	0.789	0.777
cumperc_all	0.877	0.846	0.826	0.796	0.798	0.796	0.786	0.779

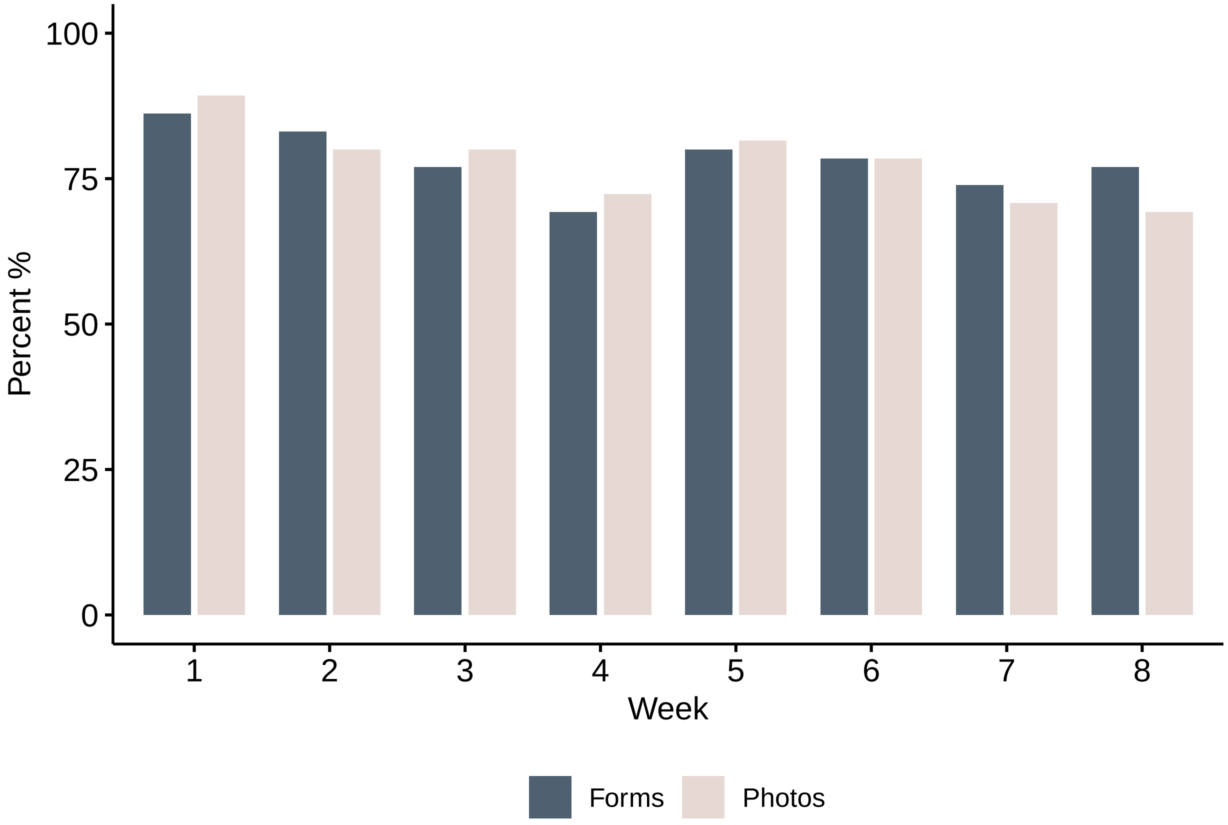

Bar plot 55 patients

variable	week_1	week_2	week_3	week_4	week_5	week_6	week_7	week_8
sum_form	52	53	50	45	52	51	48	50
sum_pic	55	51	52	47	53	51	46	45
n	55	55	55	55	55	55	55	55
sum_all	107	104	102	92	105	102	94	95
n_all	110	110	110	110	110	110	110	110
perc_form	0.945	0.964	0.909	0.818	0.945	0.927	0.873	0.909
perc_pic	1	0.927	0.945	0.855	0.964	0.927	0.836	0.818
cumsum_form	52	105	155	200	252	303	351	401
cumsum_pic	55	106	158	205	258	309	355	400
cumsum_n	55	110	165	220	275	330	385	440
cumsum_all	107	211	313	405	510	612	706	801
cumsum_n_all	110	220	330	440	550	660	770	880
cumperc_form	0.945	0.955	0.939	0.909	0.916	0.918	0.912	0.911
cumperc_pic	1	0.964	0.958	0.932	0.938	0.936	0.922	0.909
cumperc_all	0.973	0.959	0.948	0.92	0.927	0.927	0.917	0.91

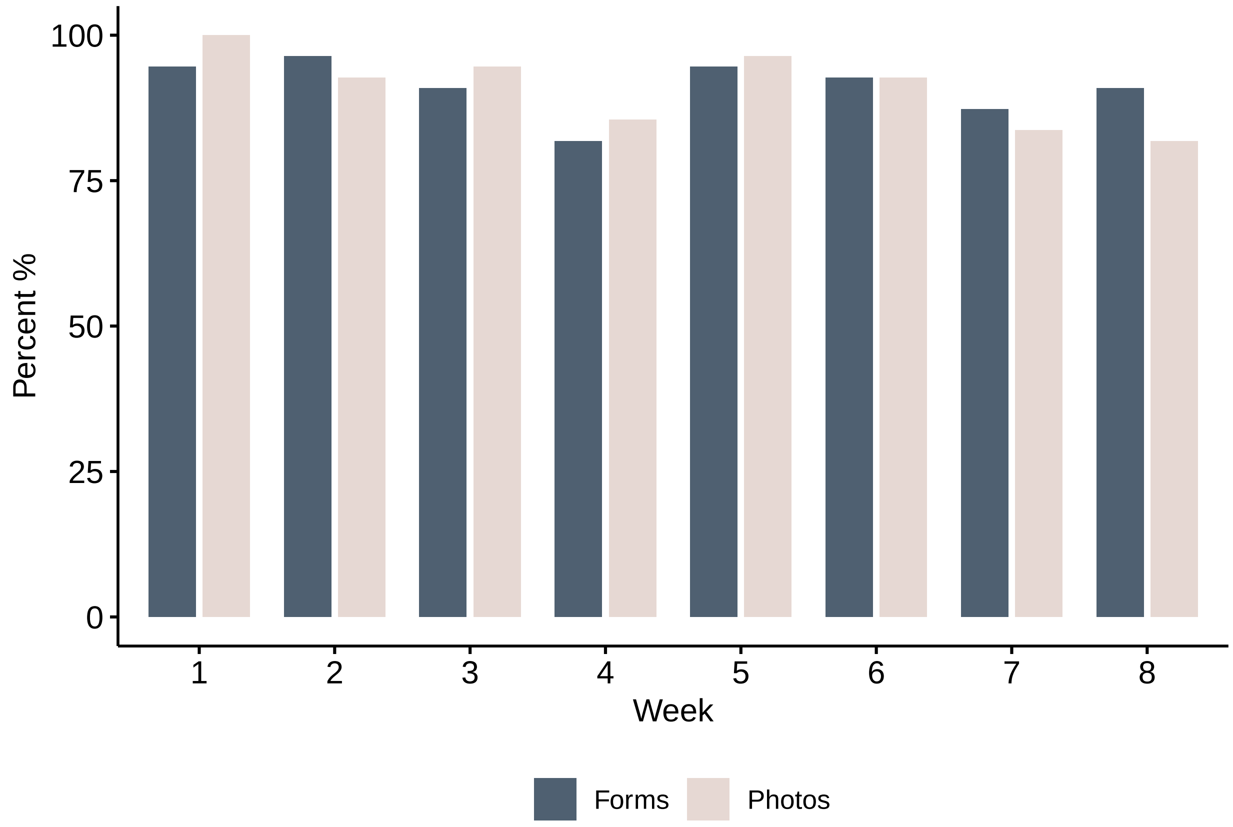

Line plots 65 patients

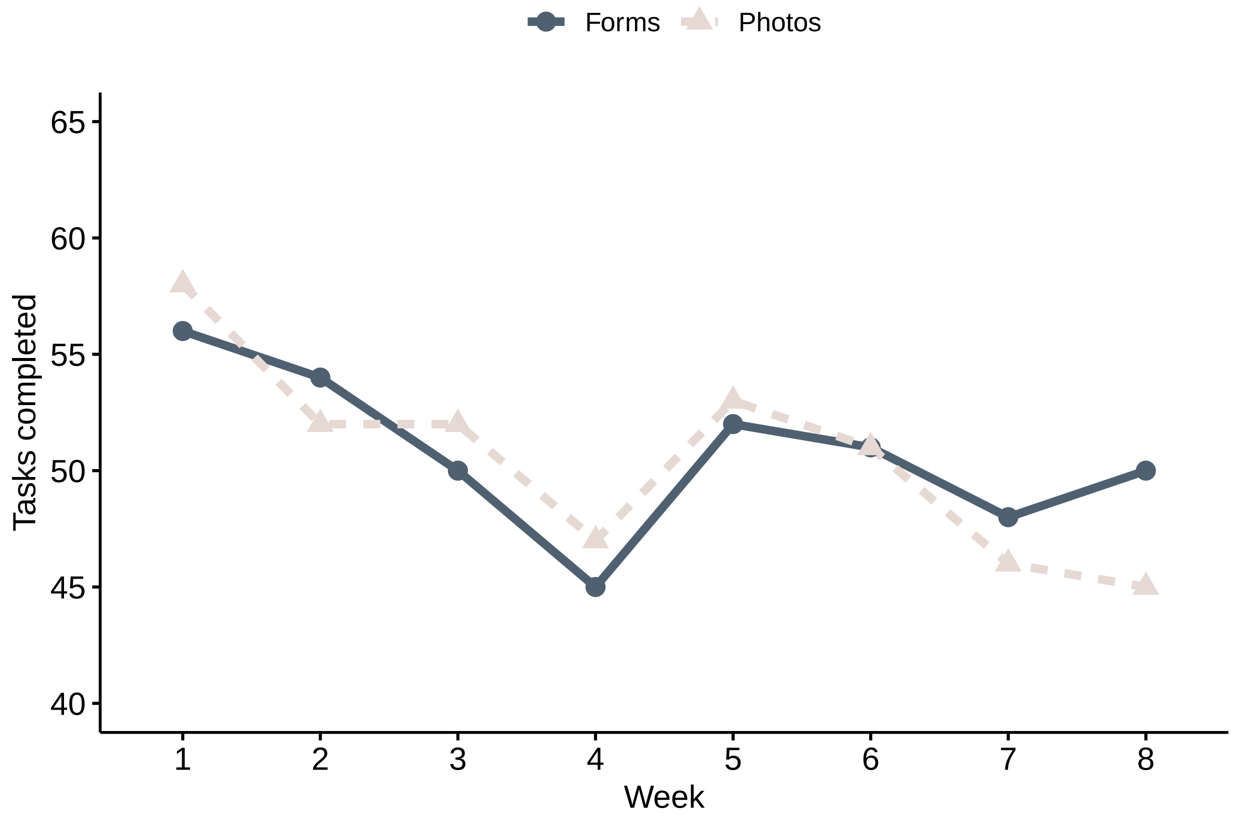

Line plots 55 patients

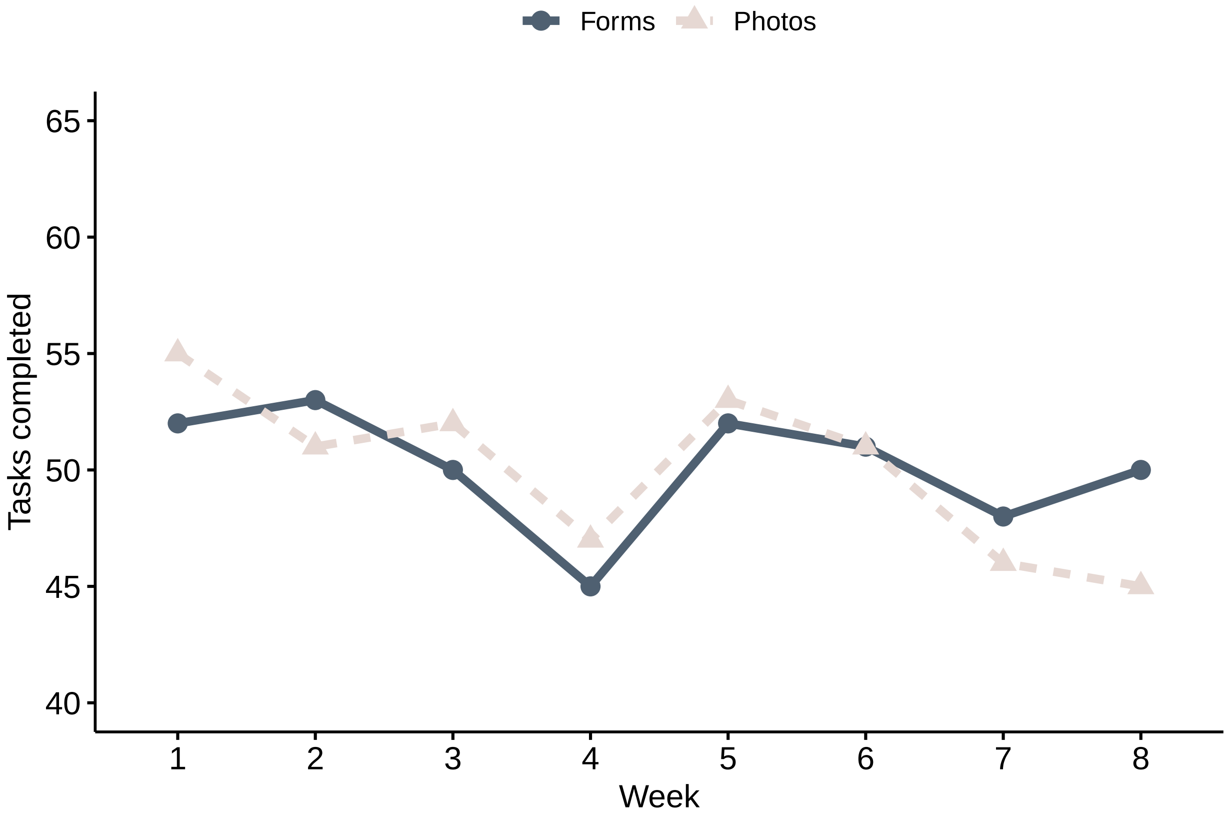

Supplement: S1 Data — (PDF) [file pone.0235500.s001.pdf]
