# Supplementary material for: High adherence and low dropout rate in a virtual clinical study of atopic dermatitis through weekly reward-based personalized genetic lifestyle reports
Source: PLoS One. 2020 Jul 2;15(7):e0235500. doi: 10.1371/journal.pone.0235500 (PMC7332076; doi:10.1371/journal.pone.0235500)
Supplement: S2 Data — (PDF) [file pone.0235500.s002.pdf]

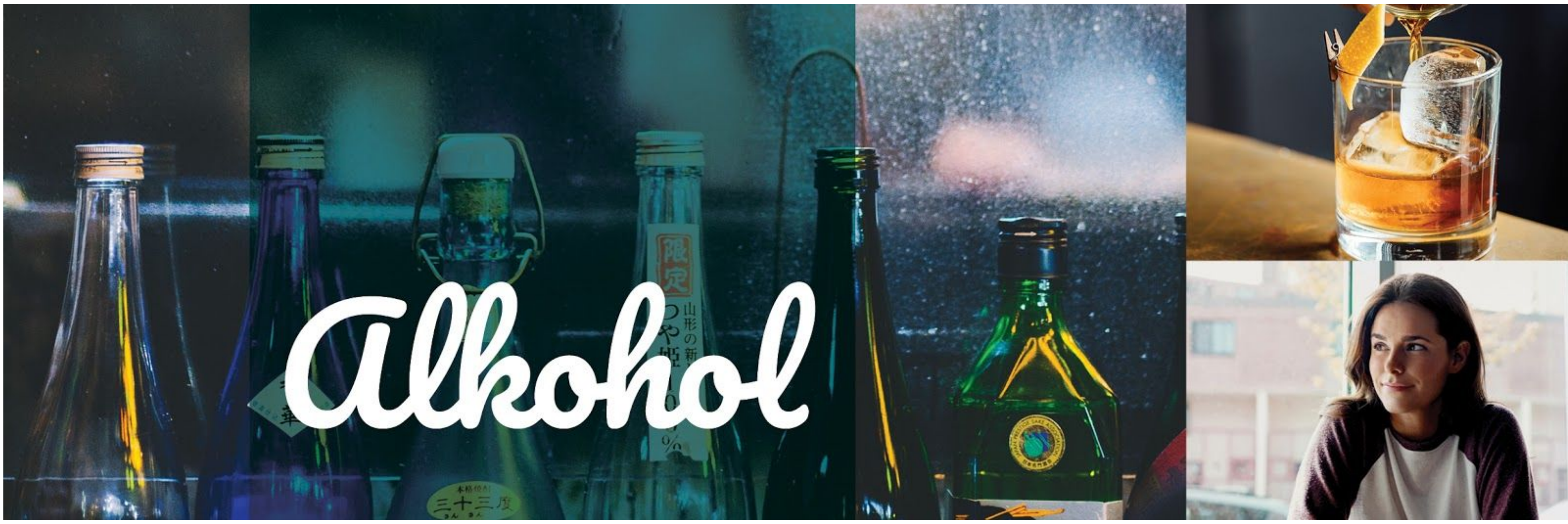

DIT RESULTAT: Sandsynlighed for høj alkohol tolerance og lav risiko for alkohol rødme
DIN HANDLING: Overvej at drikke mindre og langsommere

Dine gen-resultater indikerer at din krop har en forøget tendens til at forbrænde alkohol effektivt og hurtigt. Det betyder at du måske har en større sandsynlighed for at drikke mere end andre, for at opnå en effekt af alkohol.

Dine gen-resultater viser også, at du måske har mindre sandsynlighed for at opleve "alkohol rødme", hvilket ellers kan afholde nogen fra at drikke. Det betyder, at du kan have større tendens til at drikke mere og mindre tendens til at få det dårligt imens du drikker. Det betyder også at du måske har mindre sandsynlighed for at opleve svære tømmermænd.

Samlet set peger dine resultater på, at du kan have en generel risiko for at drikke for meget. For at forblive sund bør du derfor tænke over dine drikkevaner og generelt overveje at drikke mindre og dermed mindske dit indtag af alkohol.

Gen	Fulde navn	DNA resultat	Effekt
ADH1B * *	Alcohol dehydrogenase 1B	AA AG **GG**	Associeret med normal alkohol forbrænding
ALDH2 * * *	Aldehyde dehydrogenase 2 family member	AA AG **GG**	Associeret med ingen alkohol rødme

HVAD ER ALKOHOL?

Alkohol er også kendt som ethanol og bliver produceret gennem fermentering. Alkohol er et rusmiddel og der er ingen ernæringsmæssige fordele ved at drikke alkohol. Alkohol dulmer overførslen af signaler til hjernen og fordi alkohol, i lave doser, har en behagelig effekt, er det den mest udbredte form for rusmiddel på verdensplan (1).

Effekten ved at drikke alkohol:

Lave doser:

- Humør skifte og eufori
- Færre hæmninger
- Mindre angst

Høje doser:

- Fuldhed
- Kvalme og opkast
- Forvirring
- Svimmelhed
- Tab af finmotorik (fx. sløret tale og besværet gang)

Farlige doser:

- Alkoholforgiftning
- Koma
- Vejrtrækning stopper
- Død

NEDBRYDNING AF ALKOHOL I KROPPEN

Alkohol og dets biprodukter er giftige og derfor forsøger din krop at nedbryde alkohol så hurtigt som muligt. Nedbrydningen af alkohol involverer to enzymer, alkohol dehydrogenase (ADH enzym) og aldehyde dehydrogenase (ALDH enzym). Enzymer hjælper en reaktion med at ske hurtigt i din krop.

Først nedbrydes alkohol af ADH til det giftige acetaldehyd, hvilket derefter nedbrydes af ALDH til uskadelig vand og kuldioxid, der nemt kan udskilles (2)(3). Som du kan se fra tabellen på den første side, har vi i din DNA set på netop de to enzymer og en lille ændring i et af disse gener, kan resultere i at et enzym virker mindre effektivt, hvilket gør det sværere at nedbryde alkohol (4)(5).

HVORFOR FÅR DU TØMMERMÆND

- **Acetaldehyd:** Er et biprodukt ved nedbrydningen af alkohol (2). Kilder peger på, at dette enzym kan være skyld i de symptomerne man oplever ved tømmermænd (6).

- **Biprodukter ved fermentation:** Udover alkohol, er der nogle biprodukter der bliver produceret under fermentering, som medvirker til smag, aroma og farve på den alkohol du drikker. Disse biprodukter antyder at forhøje risikoen for værre tømmermænd (7). Der er eksempelvis flere af disse biprodukter i rom og rødvin og derfor giver disse alkoholiske drikke værre tømmermænd. Samtidig er der færre biprodukter i lyse alkoholiske drikke, som eksempelvis øl og gin, hvilket siges at give færre tømmermænd (8).

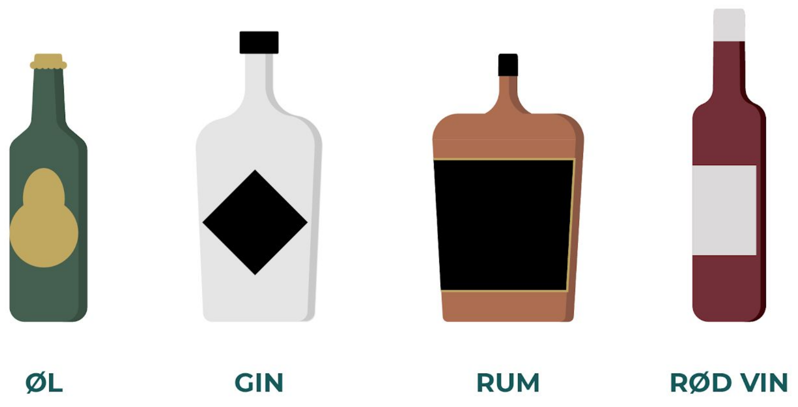

- **Langsommere hjerneaktivitet:** Tømmermændsramte personer har en langsommere hjerneaktivitet, målt på hjernens elektriske aktivitet (10). Den langsomme hjerneaktivitet forbliver i op til 16 timer, efter alkoholen ikke længere kan registreres i blodet.

- **Dehydrering:** Alkohol hæmmer udskillelsen af hormonet antidiuretisk hormon (ADH) fra hypofysen i hjernen. ADH er et hormon der fortæller nyrerne at de skal absorbere vand. Når ADH hæmmes, er der lavere niveauer af ADH, og nyrerne absorbere derfor mindre vand, hvilket resultere i dehydrering og hyppig vandladning (7).

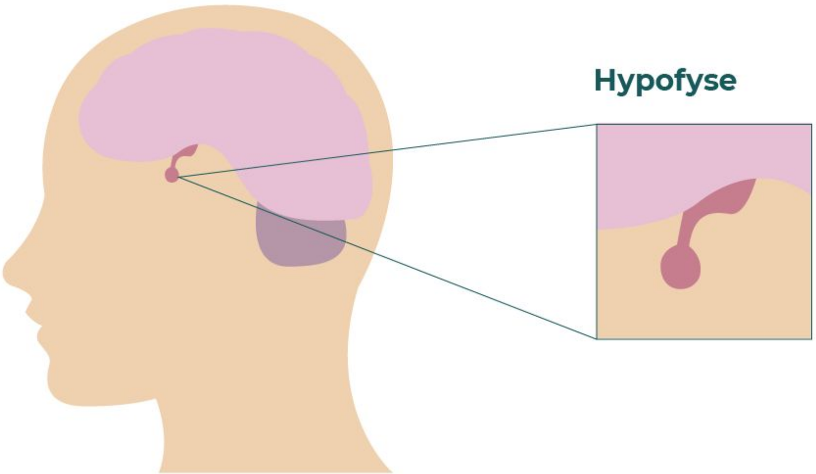

SUNDHEDSPROBLEMER OG ALKOHOL

Et stort alkoholindtag indtag over en længere periode er associeret med mange sundhedsproblemer (9). En af disse problemer er, at alkohol skader dine celler. Alkohol forårsager oxidativ stress, som er en skade inde i dine celler, som kan føre til at din DNA bliver skadet. Selvom DNA ofte kan reparere sig selv, kan vedvarende skade på din DNA føre til permanent skade. Dette er grunden til at alkoholindtag er blevet associeret med højere risiko for at udvikle kræft (10). Desuden er alkohol et rusmiddel man kan blive afhængig af, hvilket betyder der er en risiko for at blive afhængig, når man kontinuerligt drikker store mængder. I Europa er forekomsten af alkoholafhængighed ca 8-16% blandt voksne (11, 12).

HVAD ER ALKOHOL RØDME

Alkohol rødme får en person til at blive rød og varm i ansigtet, på halsen og skuldrene efter at have indtaget små mængder alkohol. Dette sker på grund af en variant i ALDH2 genet, hvor G er skiftet til A (se første side), hvilket fører til mindre effektiv nedbrydning af alkohol. Dette resulterer i, at det giftige biprodukt acetaldehyd kan udløse en respons i kroppen, der resulterer i alkohol rødme (Alcohol Flush på engelsk).

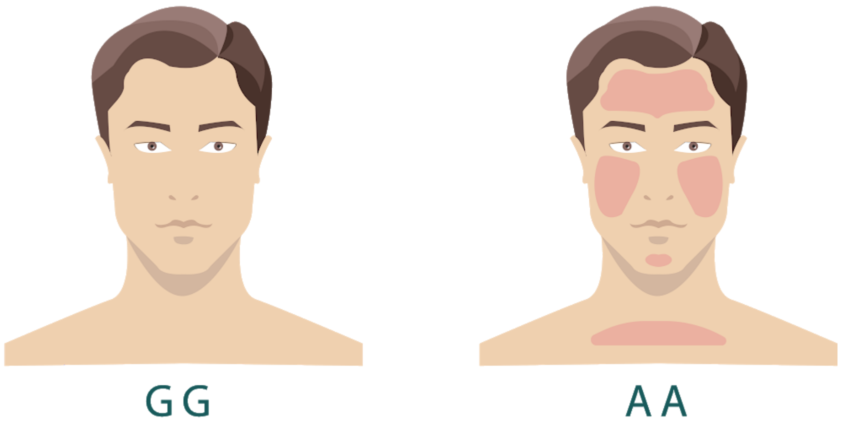

Derudover er denne variant i ALDH2 genet også blevet associeret med værre tømmermænd (13). Denne variant er utrolig sjælden i den Europæiske befolkning. Varianten ses dog hyppigere i den Øst Asiatiske befolkning, hvor op til 17% har denne variant. Varianten ses oftest i den Sydlige Han Kinesiske befolkning med en forekomst på 27% og i den Japanske befolkning med en forekomst på 24% (14).

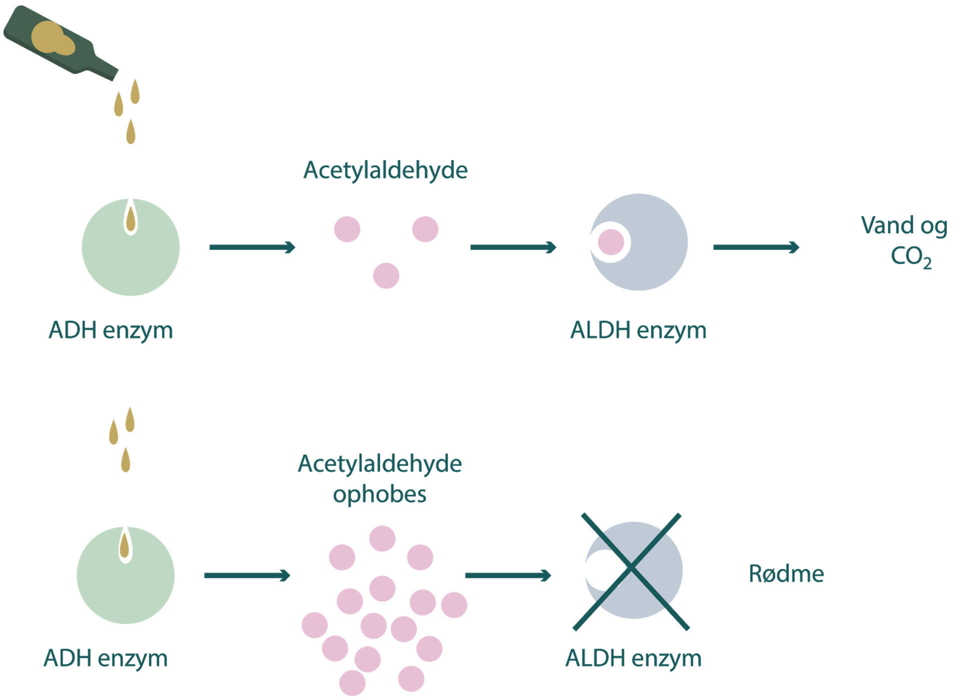

ALKOHOL FERMENTERING

Fermentering er en naturlig process, der kræver, gær, sukker og varme. Frugt der gror på træer har levende gær på deres yderside og når frugten er moden og ydersiden er gået i stykker, da kan gæren komme ind i frugten og ændre frugtsukkeret til alkohol. Mennesker er ikke de eneste der nyder alkohol, mange dyr spiser fermenteret frugt og nogen er specielt adapteret til at spise denne frugt uden at opleve negative effekter fra alkoholen. Et eksempel på et sådan dyr er frugt flagermusen (15,16).

HISTORIE

- **Alkohol fermentering er blevet dyrket af mennesker i flere tusind år:**
- **Tidligste evidens på at mennesker har lavet alkohol er fundet i det nordlige Kina mellem 7000 og 6000 f.kr. (17).**
- **I det gamle Egypten blev øl set som gudernes drik (18)**
- **I det gamle Egypten og Grækenland blev alkohol drukket som medicin. Desuden var vin så vigtig for de gamle grækere, at de havde en vingud - Dionysus (19).**
- **Under kolonitiden var øl ofte englændernes foretrukne drink, da de ikke var vant til at drikke vand (20).**
- **I dag har ca. 10.9 millioner voksne, over 26 år, et alkoholmisbrug (ca. 5.2% af den voksne population) (21)(22).**

Vær venligst opmærksom på at disse resultater er baseret på associations studier. Dette giver os en sandsynlighed for hvordan kroppen reagerer på alkohol, men dette kan også være influeret af mange andre faktorer. Bemærk venligst, at hvis du er i tvivl om du måske selv har et problem med alkohol, bør du tage kontakt til din egen læge, som vil kunne hjælpe dig.

References

1. GBD 2016 Alcohol Collaborators. Alcohol use and burden for 195 countries and territories, 1990-2016: a systematic analysis for the Global Burden of Disease Study 2016. Lancet. 2018 Sep 22;392(10152):1015–35.
2. Edenberg HJ. The genetics of alcohol metabolism: role of alcohol dehydrogenase and aldehyde dehydrogenase variants. Alcohol Res Health. 2007;30(1):5–13.
3. Alcohol Metabolism-Alcohol Alert No.35-1997 [Internet]. [cited 2019 May 2]. Available from: https://pubs.niaaa.nih.gov/publications/aa35.htm
4. Macgregor S, Lind PA, Bucholz KK, Hansell NK, Madden PAF, Richter MM, et al. Associations of ADH and ALDH2 gene variation with self report alcohol reactions, consumption and dependence: an integrated analysis. Hum Mol Genet. 2009 Feb 1;18(3):580–93.
5. Yoshida A, Huang IY, Ikawa M. Molecular abnormality of an inactive aldehyde dehydrogenase variant commonly found in Orientals. Proc Natl Acad Sci U S A. 1984 Jan;81(1):258–61.
6. Bogin RM, Nostrant TT, Young MJ. Propranolol for the treatment of the alcoholic hangover. Am J Drug Alcohol Abuse. 1987;13(1-2):175–80.
7. Wiese JG, Shlipak MG, Browner WS. The alcohol hangover. Ann Intern Med. 2000 Jun 6;132(11):897–902.
8. Chapman LF. Experimental induction of hangover. Q J Stud Alcohol. 1970 May;5:Suppl 5:67–86.
9. WHO | Alcohol. 2019 Apr 11 [cited 2019 May 2]; Available from: https://www.who.int/topics/alcohol_drinking/en/
10. Alkohol og kræft - Kræftens Bekæmpelse [Internet]. Kræftens Bekæmpelse. [cited 2019 May 2]. Available from: https://www.cancer.dk/forebyg/drik-mindre-alkohol/alkohol-og-kraeft/
11. Rehm J, Allamani A, Elekes Z, Jakubczyk A, Manthey J, Probst C, et al. Alcohol dependence and treatment utilization in Europe - a representative cross-sectional study in primary care. BMC Fam Pract. 2015 Jul 29;16:90.
12. Prevalence of alcohol use disorders [Internet]. World Health Organization. Available from: https://www.who.int/gho/substance_abuse/burden/alcohol_prevalence_text/en/
13. Yokoyama M, Yokoyama A, Yokoyama T, Funazu K, Hamana G, Kondo S, et al. Hangover susceptibility in relation to aldehyde dehydrogenase-2 genotype, alcohol flushing, and mean corpuscular volume in Japanese workers. Alcohol Clin Exp Res. 2005 Jul;29(7):1165–71.
14. rs671 (SNP) - Population genetics - Homo sapiens - Ensembl genome browser 96 [Internet]. [cited 2019 May 2]. Available from: http://www.ensembl.org/Homo_sapiens/Variation/Population?db=core;r=12:111803462-111804462;v=rs671;vdb=variation;vf=471574358
15. Wiens F, Zitzmann A, Lachance M-A, Yegles M, Pragst F, Wurst FM, et al. Chronic intake of fermented floral nectar by wild treeshrews. Proc Natl Acad Sci U S A. 2008 Jul 29;105(30):10426–31.
16. Orbach DN, Veselka N, Dzal Y, Lazure L, Fenton MB. Drinking and flying: does alcohol consumption affect the flight and echolocation performance of phyllostomid bats? PLoS One. 2010 Feb 1;5(2):e8993.
17. Gately I. Drink: A Cultural History of Alcohol. Penguin; 2008. 560 p.
18. Mark JJ. Grave Goods in Ancient Egypt [Internet]. Ancient History Encyclopedia. [cited 2019 May 2]. Available from: https://www.ancient.eu/article/1049/grave-goods-in-ancient-egypt/
19. Rosso AM. Beer and wine in antiquity: beneficial remedy or punishment imposed by the Gods? Acta Med Hist Adriat. 2012;10(2):237–62.
20. Drinking in Colonial America: Rattle-Skull, Stonewall, Bogus, Blackstrap, Bombo, Mimbo, Whistle Belly, Syllabub, Sling, Toddy, and Flip [Internet]. [cited 2019 May 2]. Available from: https://www.history.org/Foundation/journal/Holiday07/drink.cfm
21. RESULTS FROM THE 2017 NATIONAL SURVEY ON DRUG USE AND HEALTH: DETAILED TABLES [Internet]. Available from: https://www.samhsa.gov/data/sites/default/files/cbhsq-reports/NSDUHDetailedTabs2017/NSDUHDetailedTabs2017.pdf
22. Authors S. Key Substance Use and Mental Health Indicators in the United States: Available from: https://www.samhsa.gov/data/sites/default/files/cbhsq-reports/NSDUHFFR2017/NSDUHFFR2017.pdf
