# Supplementary material for: High adherence and low dropout rate in a virtual clinical study of atopic dermatitis through weekly reward-based personalized genetic lifestyle reports
Source: PLoS One. 2020 Jul 2;15(7):e0235500. doi: 10.1371/journal.pone.0235500 (PMC7332076; doi:10.1371/journal.pone.0235500)
Supplement: S3 Data — (PDF) [file pone.0235500.s003.pdf]

Feedback questionnaire		
**Danish text**	**English translation**	**Source**
Hvilke ord synes du bedst beskriver rapporterne? *(sæt gerne kryds ved flere: Tydelige, Rodet, Spændende, Kedelige, Flotte, Troværdige, Videnskabelige, Intetsigende, Other)*	Which words do you think best describe the reports? *(please tick several: Clear, Messy, Exciting, Boring, Beautiful, Credible, Scientific, Bland, Other)*	
Læste du al teksten i DNA-rapporterne? *(Slet ikke, Kun overskrifterne, Jeg sprang nogle sektioner over, Jeg læste det meste, Jeg læste alt, Andet)*	Did you read all the text in the DNA reports? *(Not at all, Only the headlines, I skipped some sections, I read most, I read everything, other)*	
Lærte du noget nyt fra rapporterne? *(Ja, Nej, Andet)*	Did you learn anything new from the reports? *(Yes, No, Other)*	
Vil du gerne have haft der var flere detaljer i DNA-rapporterne? *(Ja, Nej, Andet)*	Would you like to have had more details in the DNA reports? *(Yes, No, Other)*	
Synes du at dine DNA resultater stemmer overens med din egen oplevelse? *(Ja, det hele matchede, Det meste passede på mig, Noget af det passede, Det meste passede ikke, Nej, jeg genkendte ikke mig selv i rapporterne)*	Do you think your DNA results match your own experience? *(Yes, it all matched, Most of it fitted to me, Some of it did fit, Most didn't fit, No, I didn't recognize myself in the reports)*	
Har du lavet nogle livsstilsændringer på baggrund af dine DNA-rapporter? *(Ja, i høj grad, Ja, nogle få, Nej, Andet)*	Have you made any lifestyle changes based on your DNA reports? *(Yes, to a great extent, Yes, a few, No, Other)*	
Hvad synes du om sproget i DNA-rapporterne? *(For simpelt, For kompliceret, scale 1-5)*	What do you think of the language in the DNA reports? *(Too simple, Too complicated, scale 1-5)*	
Havde du problemer med at forstå ord	phrases in the reports? *(Yes many, Yes a few, No,*	
Hjalp illustrationerne dig til at forstå de	scientific concepts? *(Yes, No, Other)*	
Hvad synes du om emnerne i de forskellige DNA-rapporter? *(Kedelig, Fint, Spændende)*	What do you think of the topics in the various DNA reports? *(Boring, fine, exciting)*	
Hvilke rapporter overraskede dig mest? (sæt gerne flere krydser) *(Gluten, D-Vitamin, Ledskader, Alkohol, Koffein, Sund vægt, Der var ikke noget der overraskede mig, Ved ikke)*	Which reports surprised you the most? (you may tick off more than one) *(Gluten, Vitamin D, Injury, Alcohol, Caffeine, Healthy Weight, None of them surprised Me, Don't know)*	
Hvilke andre livstilsemner ville du gerne have modtaget DNA-rapporter om?	What other lifestyle topics would you like to receive DNA reports on?	
Jeg oplevede at DNA-rapporterne motiverede mig til at udføre mit ugentlige check-in (spørgeskema og billeder): *0 (unenig) - 5 (enig)*	I found that the DNA reports motivated me to do my weekly check-in (questionnaire and pictures): *0 (disagree) - 5 (agree)*	
Jeg oplevede at de ugentlige påmindelser og notifikationer var en hjælp til at udføre mit check-in: *0 (unenig) - 5 (enig)*	I found that the weekly reminders and notifications were helpful in performing my check-in: *0 (disagree) - 5 (agree)*	
Jeg satte pris på den ugentlige "Vidste du" fact i mine e-mails: *0 (unenig) - 5 (enig)*	I appreciated the weekly "Did you know" fact in my emails: *0 (disagree) - 5 (agree)*	

Hvilken anden form for påmindelse ville du gerne have modtaget? *(SMS, E-mail, Notifikation i en App, Telefonopkald, Ingen anden påmindelserne fungerede fint, Andet)*	What other kind of reminder would you like to receive? *(SMS, Email, Notification in an App, Phone call, Nothing the reminders worked fine, Other)*		
Missede du et ugentlig check-in (spørgeskema eller billeder) undervejs i undersøgelsen? - Hvis ja, hvad var så årsagen til dette? *(vælg gerne flere: Travlhed, Glemte det, Ferie, Sygdom, Mistede interessen, Det kan jeg ikke huske, Jeg glemte ikke nogen)*	Did you miss a weekly check-in (questionnaire or pictures) during the survey? - If so, what was the reason for this? *(please select multiple: Busy, Forgotten it, Holidays, Illness, Lost interest, I can't remember, I didn't forget any)*		
Hvad skulle der til for at du ikke missede en eneste af dine ugentlige check-in?	What did it take for you not to miss a single one of your weekly check-ins?		
Hvad motiverede dig til at udføre dit ugentlige check-in?	What motivated you to do your weekly check-in?		
Nu hvor du har færdiggjort undersøgelsen, vil du så gøre det igen (fx. for flere eller andre rapporter)? *(Ja, Nej, Andet)*	Now that you have completed the survey, do you want to do it again (eg for more or other reports)? *(Yes, No, Other)*		
Vil du anbefale undersøgelsen til andre? *(Ja, Nej, Andet)*	Would you recommend the study to others? *(Yes, No, Other)*		
Har du nogen anden feedback til denne undersøgelse?	Do you have any other feedback for this study?		
Må vi ringe til dig og følge op på din oplevelse med MyImagineDNA? *(Ja, Nej tak)*	May we call you and follow up on your experience with MyImagineDNA? *(Yes, No thanks)*		
Har du taget en DNA test før? *(Ja hos min læge, Ja på egen opfordring, Nej)*	Have you taken a DNA test before? *(Yes at my doctor, Yes at my own request, No)*		
Har du følt dig komfortabel ved at dele din DNA data med MyImagineDNA? *(Ja meget, Ja lidt, Nej ikke rigtig, Nej, Andet)*	Have you felt comfortable sharing your DNA data with MyImagineDNA? *(Yes very, Yes quite, No not reallty, No, other)*		
Havde du nogle problemer med at udføre DNA testen? *(Ja, Nej, Andet)*	Did you have any problems performing the DNA test? *(Yes, No, Other)*		
Havde du nogen problemer i forbindelse med at sende din DNA test afsted? *(Ja, Nej, Andet)*	Did you have any problems sending your DNA test? *(Yes, No, Other)*		
Jeg oplevede at hjemmesiden (www.myimaginedna.com) var tydelig og havde den information jeg skulle bruge. *1 (Uenig) - 5 (Enig)*	I found that the website (www.myimaginedna.com) was clear and had the information I needed. *1 (Disagree) - 5 (Agree)*		
Svarer den information, der fremgik på hjemmesiden til din oplevelse af at deltage i undersøgelsen? *1 (Nej) - 5 (Ja)*	Does the information provided on the website correspond to your experience of participating in the survey? *1 (No) - 5 (Yes)*		
Vendte du tilbage til hjemmesiden under undersøgelsen for at få mere information? *(Ja mange gang, Ja et par gang, Nej, Andet)*	Did you return to the website during the investigation for more information? *(Yes many times, Yes a couple of times, No, Other)*		
Hvad fik dig til at tilmelde dig undersøgelsen?	What prompted you to sign up for the survey?		
Jeg synes at den tid det krævede af mig at udføre mit ugentlige check-in var rimelig. *1 (Ikke rimelig) - 5 (Rimelig)*	I think the time it took for me to do my weekly check-in was reasonable. *1 (Not reasonable) - 5 (reasonable)*		

Stemte den tid du brugte på at udføre dit check-in overens med dine forventninger om, hvor lang tid det ville tage? *1 (Mindre) - 5 (Mere)*	Did the time you spend completing your check-in match your expectations of how long it would take? *1 (Less) - 5 (More)*	
Ville du have færdiggjort undersøgelsen, hvis den havde været længere? - og hvis ja, hvor meget længere? *(Ja i 12 uger, Ja i 6 måneder, Ja i 1 år, Nej jeg har ikke færdiggjort undersøgelsen hvis den havde været længere)*	Would you have completed the study if it had been longer? - and if so, how much longer? *(Yes for 12 weeks, Yes for 6 months, Yes for a year, No I wouldn't have continued the study if it had been longer)*	
Imagine appen var simpel at sætte up og bruge *(Ja, Nej, Andet)*	The Imagine app was simple to set up and use *(Yes, No, Other)*	
Brugte du notifikationerne i din Imagine app til at tage dine ugentlige billeder? *(Ja jeg brugte dem til at huske mig på at tage billede hver uge, Ja men e-mail påmindelserne var bedre, Nej jeg brugte dem ikke)*	Did you use the notifications in your Imagine app to take your weekly photos? *(Yes I used them to remind me to take pictures every week, Yes but the email reminders were better, No I didn't use them)*	
Hvor lang tid tog det dig at udfylde det ugentlige spørgeskema? *(Mindre end 5 minutter, 5-1 minutter, Mere end 10 minutter)*	How long did it take you to complete the weekly questionnaire? *(Less than 5 minutes, 5-10 minutes, More than 10 minutes)*	
Havde du nogen problemer i forbindelse med at udfylde spørgeskemaet? - og i så fald hvilke?	Did you have any problems completing the questionnaire? - and if so, which ones?	
Det ugentlige spørgeskema var.. For	too long	
Vil du mene at de sidste 8 uger har været repræsentativ for din hud? *(Meget repræsentativ, Rimelig repræsentativ, Ikke ret repræsentativ, Slet ikke repræsentativ)*	Do you think the last 8 weeks have been representative of your skin? *(Very representative, Fairly representative, Not quite representative, Not at all representative)*	
Er du interesseret i at donere den DNA data, vi har indsamlet om dig til fremtidig forskning? (Hvis du er interesseret, vil du modtage yderligere information før du træffer din endelig beslutning. Du vil altid kunne kræve din data slettet) *(Jeg vil gerne høre mere om at donere min DNA til forskning, Jeg vil ikke donere min DNA til fremtidig forskning)*	Are you interested in donating the DNA data we have collected about you for future research? (If you are interested, you will receive additional information before making your final decision. You will always be able to claim your data deleted) *(I would like to hear more about donating my DNA for research, I will not donate my DNA for future research)*	
Må vi beholde din kontaktinformation? Vi vil udelukkende skrive til dig, hvis vi har et fremtidigt studie omkring eksem, hvor vi leder efter deltagere. *(Ja, Nej, Andet)*	May we keep your contact information? We will only write to you if we have a future eczema study where we are looking for participants. *(Yes, No, Other)*	
Bekræft venligt din e-mail adresse	Please confirm your email address	
